# Microglial cell response to experimental periodontal disease

**DOI:** 10.1186/s12974-023-02821-x

**Published:** 2023-06-14

**Authors:** Rawan Almarhoumi, Carla Alvarez, Theodore Harris, Christina M. Tognoni, Bruce J. Paster, Isabel Carreras, Alpaslan Dedeoglu, Alpdogan Kantarci

**Affiliations:** 1 Forsyth Institute, 245 First Street, Cambridge, MA 02142 USA; 2 Department of Oral Medicine, Infection, and Immunity, Harvard School of Dental Medicine, Boston, MA 02115 USA; 3 Faculty of Dentistry, King Abdulaziz University, Jeddah, Saudi Arabia; 4 Department of Veterans Affairs, VA Boston Healthcare System, Research and Development Service, Building 1A-(151), 150 S. Huntington Avenue, Boston, MA 02130 USA; 5 Department of Neurology, Boston University School of Medicine, Boston, MA 02118 USA; 6 Department of Biochemistry, Boston University School of Medicine, Boston, MA 02118 USA; 7 Department of Radiology, Massachusetts General Hospital and Harvard Medical School, Boston, MA 02114 USA

**Keywords:** Microglia, Neuroinflammation, Alzheimer's disease, Periodontal disease, Microbiology

## Abstract

**Objectives:**

Microglial activation is critical for modulating the neuroinflammatory process and the pathological progression of neurodegenerative diseases, such as Alzheimer's disease (AD). Microglia are involved in forming barriers around extracellular neuritic plaques and the phagocytosis of β-amyloid peptide (Aβ). In this study, we tested the hypothesis that periodontal disease (PD) as a source of infection alters inflammatory activation and Aβ phagocytosis by the microglial cells.

**Methods:**

Experimental PD was induced using ligatures in C57BL/6 mice for 1, 10, 20, and 30 days to assess the progression of PD. Animals without ligatures were used as controls. Maxillary bone loss and local periodontal tissue inflammation associated with the development of PD were confirmed by morphometric bone analysis and cytokine expression, respectively. The frequency and the total number of activated microglia (CD45^+^ CD11b^+^ MHCII^+^) in the brain were analyzed by flow cytometry. Mouse microglial cells (1 × 10^5^) were incubated with heat-inactivated bacterial biofilm isolated from the ligatures retrieved from the teeth or with *Klebsiella variicola*, a relevant PD-associated bacteria in mice. Expression of pro-inflammatory cytokines, toll-like receptors (TLR), and receptors for phagocytosis was measured by quantitative PCR. The phagocytic capacity of microglia to uptake β-amyloid was analyzed by flow cytometry.

**Results:**

Ligature placement caused progressive periodontal disease and bone resorption that was already significant on day 1 post-ligation (*p* < 0.05) and continued to increase until day 30 (*p* < 0.0001). The severity of periodontal disease increased the frequency of activated microglia in the brains on day 30 by 36%. In parallel, heat-inactivated PD-associated total bacteria and *Klebsiella variicola* increased the expression of TNFα, IL-1β, IL-6, TLR2, and TLR9 in microglial cells (1.6-, 83-, 3.2-, 1.5-, 1.5-fold, respectively *p* < 0.01). Incubation of microglia with *Klebsiella variicola* increased the Aβ-phagocytosis by 394% and the expression of the phagocytic receptor MSR1 by 33-fold compared to the non-activated cells (*p* < 0.0001).

**Conclusions:**

We showed that inducing PD in mice results in microglia activation in vivo and that PD-associated bacteria directly promote a pro-inflammatory and phagocytic phenotype in microglia. These results support a direct role of PD-associated pathogens in neuroinflammation.

**Supplementary Information:**

The online version contains supplementary material available at 10.1186/s12974-023-02821-x.

## Introduction

Chronic neuroinflammation is crucial in the pathophysiology of various neurodegenerative diseases, including Alzheimer’s Disease (AD) [1, 2]. Microglia are the resident mononuclear phagocyte population within the central nervous system (CNS), sharing phenotypical and functional characteristics with macrophages, and are essential to the brain’s immune and inflammatory response [1, 3]. Microglia are rapidly activated upon exogenous stimulation or microenvironment changes, releasing cytokines with an upregulation of major histocompatibility complex II (MHC II) molecules [1]. Microglia are also critical for phagocytosis of pathogens and debris, including amyloid beta (Aβ) protein, through class A scavenger receptor SR-A (macrophage scavenger receptor 1 MSR1), Class B scavenger receptor CD36, the receptor for advanced-glycosylation end products (RAGE), and Formyl peptide receptors (FPRs) [4–6]. These functions are regulated by CD68, CD14, CX3CR1, and toll-like receptors (TLRs) [3, 7, 8]. TLRs are associated with microglial recognition of patterns on bacterial pathogens, where CD14 acts as a co-receptor for transmembrane TLR2 and TLR4, presenting antigens to them [3, 9]. Downstream signaling in microglial cells is mediated by Nuclear Factor kappa-light-chain-enhancer of activated B cells (NF-κB) activation and the transcription of pro-inflammatory genes (e.g., TNF-α, IL-1α, IL-6) [8]. Thus, microglia are the multi-tasking first line of defense in the brain, orchestrating the inflammatory response upon detecting any danger signals presented by infectious stimuli or debris [3].

Periodontal disease (PD), or periodontitis, is an oral inflammatory disease caused by the host’s immunological response to periodontal pathogens [10]. PD is a significant public health burden with a high prevalence among adults. 42% of US adults aged 30 or older have periodontitis, with 7.8% having severe forms of the disease. Periodontitis prevalence increases with age, affecting more than 59% of adults over 65 [11, 12]. Chronic PD is a slow, progressive, irreversible degradation of the tooth-supporting apparatus (periodontium), leading to tooth loss. [13, 14]. PD is initiated by dysbiosis of the microbiome of the oral cavity and results in local inflammation, eventually contributing to chronic systemic inflammation [10, 15]. Periodontitis is associated with neurodegenerative diseases and neuroinflammatory processes through circulating mediators or direct access of the oral microbes to the CNS via systemic circulation [16–18]. Different species of *Treponema* associated with periodontitis were detected in the brains of AD cases [19]. Lipopolysaccharide (LPS) and gingipains of *Porphyromonas gingivalis*, a prominent PD-associated bacterial species in humans, were also detected in the brains of AD patients [20, 21]. In mice, neuroinflammation and amyloid plaque formation developed after repeated oral application of *P. gingivalis* [22], suggesting that both infectious and inflammatory mechanisms are plausible in the link between PD and neurodegenerative processes. However, whether PD-associated pathogens can directly activate the microglial function is unclear. The ligature-induced model of PD does not need human bacteria; it is induced by commensal and native bacteria. In this study, we tested the hypothesis that PD-associated pathogens impact inflammatory activation and Aβ phagocytosis by microglial cells.

## Materials and methods

### Animals and experimental groups

**Fig. 1:**
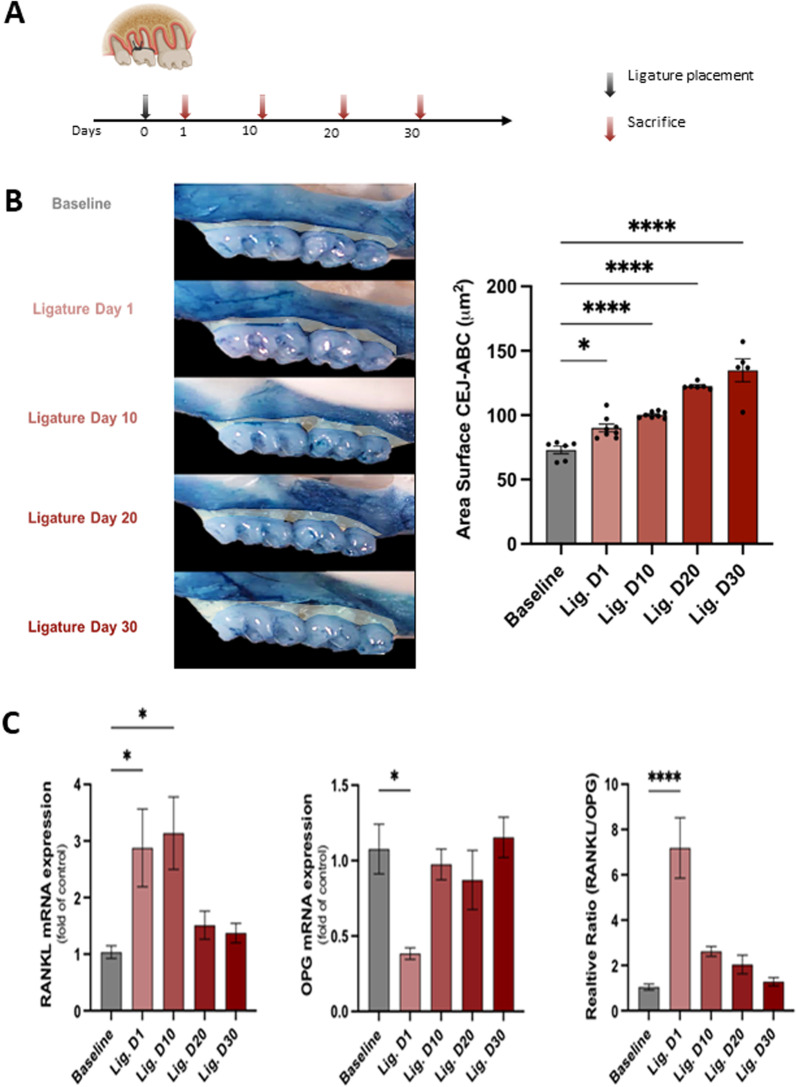
Alveolar bone resorption and mRNA expression levels of RANKL and OPG in periodontal tissues of mice with ligature-induced experimental periodontal disease. **A** In vivo experimental design (*n* = 36, 7–8 mice per group). The baseline group was left untreated, and four groups had ligatures around maxillary right and left second molars for different timepoints corresponding to 1,10, 20, and 30 days and then sacrificed. Black arrow: placement of ligatures. Red arrows: sacrifice. **B** Left: representative images of the left maxillae from each experimental group (buccal view). Right: quantification of the area between the alveolar bone crest level and the cementoenamel junction of the three maxillary molars, using Fiji software (ImageJ). **C** Levels of mRNA expression of proteins involved in periodontal tissue metabolism RANKL and OPG. The right and left gingival tissue of each mouse were pooled together and represented one sample. Baseline: mice without ligature placement. Lig. D1, D10, D20, and D30: mice with ligature placed for different timepoints corresponding to 1,10, 20, and 30 days, respectively. (*n* = 36, 7–8/group, Mean ± SEM, ANOVA, *p < 0.05, ****p < 0.0001)

Thirty-six C57BL/6 wild-type, aged 10–12 weeks, were used. The mice were maintained in a specific pathogen-free environment, 12:12 h light/dark cycle at 24 ± 0.5 °C and 40–70% relative humidity. The Institutional Animal Care and Use Committee of the Forsyth Institute reviewed and approved the experimental protocols. Mice were divided into five groups of 7 to 8 mice per group, including mice with no ligature (baseline) and mice used on day 1, 10, 20, and 30 post-ligature placements, as shown in Fig. 1A. The ligatures were placed using a 7-0 silk suture subgingivally around the maxillary right and left second molars with the knot positioned at the palatal side, as previously reported [23]. Ligature placement was performed under general anesthesia using a ketamine/xylazine cocktail (87.5 mg of ketamine per kg of body weight and 12.5 mg of xylazine per kg of body weight) via intraperitoneal injection. The procedure was performed using fine microsurgical instruments, a microscope*,* a cold-light source system, and animal-holding support to allow the maximum mouth opening without causing mechanical damage to the oral mucosa of the anesthetized animal*.*

### Assessment of alveolar bone loss and periodontal inflammatory process

To confirm the alveolar bone loss as a direct assessment of experimental periodontal disease, we measured the loss of bone in defleshed mouse jaws dissected immediately after the animals were euthanized. After removing ligatures and gingival tissue from the sacrificed animals, the maxillae were defleshed by dermestid beetles for 4–5 days. Then, the samples were cleaned with 5% hydrogen peroxide for 8 h and washed thoroughly with water. The samples were stained with methylene blue (1% in water) for 10 s to clearly distinguish between the bone and the teeth before the morphometric analysis of the bone loss. Then, the samples were mounted and photographed at × 10 magnification using an inverted microscope (Axiovert 200, Zeiss, Thornwood, NY, USA) and AxioVision 4.8 software. The area between the alveolar bone crest level and the cementoenamel junction of the three maxillary molars was measured using ImageJ and calculated in micrometers.

For further confirmation of bone metabolism and the resorptive process, the gingival tissues were collected around all maxillary molars and fixed in RNAlater (Life Technologies, Carlsbad, CA, USA). Then, the samples were homogenized, and total RNA was isolated through the RNeasy® Mini kit (QIAGEN, Germantown, MD, USA) according to the manufacturer's instructions. The purity was tested using a NanoDrop ND-1000 spectrophotometer (absorbance ratio at 260 and 280 nm, ThermoFisher Scientific, Waltham, MA, USA). One µg of RNA was reverse-transcribed to cDNA through High-Capacity cDNA Reverse Transcription Kit (Applied Biosystems, ThermoFisher, Waltham, MA, USA) using RT random primers, dNTP mix, RT buffer, nuclease-free water, ribonuclease (RNase) inhibitor, and reverse transcriptase for quantitative PCR (Q-PCR) analysis using StepOnePlus Real-Time PCR system (Applied Biosystems, ThermoFisher, Waltham, MA, USA). The detection assay was done using the TaqMan™ Fast Advanced Master Mix (ThermoFisher Scientific, Waltham, MA, USA), 50 ng cDNA of gingival tissue, and TaqMan Gene Expression Assays of RANKL and OPG (Additional file 1: Table S1). Data were analyzed using the 2^−ΔΔCT^ method, and the expression of each target gene was then calculated as a fold-change relative to the controls using β-actin as an endogenous control.

### Isolation of adult mouse brain cells

Isolation of cells from the adult mouse brain was done according to a previously published protocol [24]. Briefly, mice were euthanized and transcardially perfused with cold PBS (10 mL/min). Then, each fresh brain was transferred into a 50 ml Falcon tube containing 10 mL cold 1 × HBSS (R&D Systems, Inc., Canada) and minced with a sterile No.15 scalpel blade on ice. The tissue samples were spun for 5 min at 400 × *g* at 4 °C, and the supernatant was aspirated. 10 mL of HBSS supplemented with 85 units dispase II (Sigma Aldrich, catalog number: D4693-1G) + 0.25 units DNaseI (Zymo Research, catalog number: E1010) + 2.5 units (CDU) collagenase Type I (Sigma Aldrich, catalog number: C0130) + 1 μg Nα-Tosyl-L-lysinechloromethyl ketone hydrochloride (Sigma Aldrich, catalog number: T7254) was added to the minced whole-brain tissue. The tubes were incubated in a water bath at 37 °C for 1 h. Then, the digested brain tissue with the enzyme cocktail was transferred into a 15 mL Dounce homogenizer (Sigma-Aldrich) and dissociated on ice using the large clearance pestle. The homogenized brain cell suspension was transferred to a 50 mL tube containing 5 mL 10% Fetal Bovine Serum (FBS) through the 70 µm cell strainer (Miltenyi Biotec). The cells were filtered by repeatedly washing the cell strainer with 5 mL 10% FBS. The obtained cell suspension was centrifuged for 5 min at 400 × *g* at 4 °C; the supernatant was discarded. Then, the cells were resuspended in 16 mL of 35% Percoll (Sigma-Aldrich, St. Louis, MO, USA). The sample was split equally into two 15 mL tubes, overlayed with 5 mL 1 × HBSS, and rested on ice for 5 min. The samples were spun at 45 min at 4 °C and 800 × *g* without a break to get the different density gradient layers. Finally, the supernatant, including the myelin layer, was discarded. The pelleted mixed brain cells were resuspended and prepared for flow cytometric analyses without further passaging to prevent any phenotypic changes.

### Flow cytometry and T-SNE analysis

Brain cells were washed with PBS and stained with the zombie UV™ Fixable Viability Kit (Biolegend, San Diego, CA, USA) for 30 min in the dark. The extracellular staining was performed in PBS containing 5% FBS, using the following antibodies: anti-CD45 (30-F11, Biolegend), anti-CD11b (M1/70, Biolegend), anti-CD68 (FA-11, Biolegend), anti-MHCII (M5/114.15.2, Biolegend), and anti-CX3CR1(SA011F11, Biolegend) for 30 min at 4◦C in the dark (Additional file 1: Table S2). Cells were analyzed on Attune™ NxT acoustic focusing cytometer (Invitrogen) using a sequential gating strategy according to the FSC/SSC and SSC/SSC parameters, live/dead staining, and CD45/CD11b markers. The multiparametric flow cytometry data analysis was performed using FlowJo software (CA, USA). The data were analyzed using dimensionality reduction with the t-Distributed Stochastic Neighbor Embedding (tSNE) algorithm. The tSNE algorithm computes two new derived parameters from a user-defined selection of cytometric parameters. The following workflow was used to compare samples effectively: (1) data clean up by applying manual gates to exclude doublets, debris, and dead cells from each sample. (2) Use the down-sample algorithm on the CD11b^+^ CD45^low^ gated populations of each sample to 20,000 events to significantly reduce calculation time. (3) Concatenate all samples (3 samples per experimental group) to generate a single two-dimensional data space created by tSNE. (4) Dimensionally reduce (create tSNE parameters) on the concatenated file using default settings in FlowJo, iterations 1000, perplexity 30, and learning rate (eta) 521. (5) Analysis of differential expression of microglial cell markers in different mice groups.

### Isolation and heat-inactivation of mouse microbiome associated with experimental periodontal disease

We isolated and collected the microbial species from the ligatures around the maxillary molars to characterize the direct impact of the mouse microbiome associated with PD on mouse microglial cells. To standardize the multiplicity of infection (MOI) of bacteria within and between individual experiments and avoid the live organisms exhibiting a rapid doubling time, which can quickly lead to culture overgrowth, medium exhaustion, and subsequent microglial death, we used heat-inactivated bacteria to activate microglial cells [25]. The ligatures were recovered from euthanized animals and were gently washed with PBS to remove food residue and other debris. Subsequently, the ligatures were placed in Eppendorf tubes containing Roswell Park Memorial Institute (RPMI) 1640 medium (Gibco, Billings, MT, USA) without any supplements. The bacteria were dispersed by vortexing for 2 min at 3000 rpm, followed by removing ligatures from the tubes as previously described [26]. Then, the number of bacteria in the suspension was determined spectrophotometrically. Finally, heat-inactivation of the total microbiome was prepared by heating the culture media for 30 min at 65 °C in a shaking water bath*.* The preparation was checked for sterility by plating on agar media. Heat-inactivated bacteria were stored at − 80 °C until use.

### Identification and culture of *Klebsiella variicola*

In separate experiments, using 16S rRNA gene sequencing, we identified the microbial composition of the ligatures and the brains of 5xFAD mice, a transgenic mouse model of AD, after 4 weeks of ligature-induced experimental periodontitis. We identified individual species that grew in the live cultures. *Klebsiella variicola* was the major species detected in direct cultures of both ligatures and brain specimens in 5xFAD mice, confirmed by direct brain cultures and 16S rRNA sequencing*.* We used *K. variicola* in subsequent studies to explore its impact on microglial cells as a periodontal bacteria in an AD-like mouse model. To prepare *K. variicola* for co-culture experiments with microglial cells, *K. variicola* was plated on Trypticase™ Soy Agar (TSA II™) with sheep blood plates aerobically at 37 °C. After 2–3 days of incubation, colonies were picked with a sterile metal loop and were grown in liquid Tryptic Soy Broth medium (TSB) aerobically at 37 °C for 1 day. Then, the number of bacteria in the suspension was determined spectrophotometrically. Finally, heat-inactivation of cells was prepared by heating bacteria-containing culture media for 30 min at 65 °C in a shaking water bath. The preparation was checked for sterility by plating. Heat-inactivated *K. variicola* was stored at − 80 °C until use.

### Microglial cell culture

The mouse microglial cell line BV2 (generated from primary mouse microglia transfected with a v-raf/v-myc oncogene carrying retrovirus (J2)) [27] was purchased from Biological Bank and Cell Factory (BBCF, Italy, www.iclc.it). BV2 cells were cultured in RPMI 1640 medium (Gibco), supplemented with 1% penicillin–streptomycin (Gibco) and 10% fetal bovine serum (FBS, ATLANTA biologicals). Cells were maintained at 37 °C in a 5% CO_2_ humidified atmosphere. Every 2 days, microglial cells were washed and dislodged from the surface of the flask by gentle pipetting with phosphate-buffered solution twice and transferred into a 50 mL tube. After spinning the culture medium containing cells, the supernatant was discarded, and the cell pellet was resuspended in 5 mL of culture medium. Then, 2 × 10^6^ cells were plated into new 75 cm^2^ flasks containing 15 mL prewarmed culture medium and placed in the incubator. Cells were passaged 2–3 times. All experiments were typically done on passage number 7. When cells reached 60–70% confluency, they were transferred into 24-well plates (1 × 10^5^ cells/well). q-PCR and flow cytometry were used to measure the expression level of specific genes involved in microglial activation and the phagocytic capacity of Aβ by microglial cells, respectively.

### Gene expression by microglial cells using q-PCR

To assess the effect of the whole PD-associated microbiome or *K. variicola* on the expression level of specific genes involved in microglial activation, microglial cells were seeded into 24-well plates and incubated in RPMI supplemented with 2% FBS and 1% penicillin–streptomycin for 24 h. The bacterial suspension (whole PD-associated microbiome from the ligature or *K. variicola*) was added to the microglial cells at different multiplicities of infection (MOI). After 24 h of co-incubation, total cellular RNA was isolated using the RNeasy® Mini kit (QIAGEN, Germantown, MD, USA) according to the manufacturer's instructions. The purity of RNA was tested using a NanoDrop ND-1000 spectrophotometer (ratio of absorbance at 260 and 280 nm, Thermo Scientific, Waltham, MA, USA). The detection assay was done using the TaqMan™ Fast Advanced Master Mix (ThermoFisher Scientific, Waltham, MA, USA), 5 ng cDNA of microglial cells, and TaqMan Gene Expression Assays (Additional file 1: Table S1). Data were analyzed using the 2^−ΔΔCT^ method, and the expression of each target gene was then calculated as a fold-change relative to the controls using β-actin as an endogenous control.

### Amyloid β phagocytosis by microglial cells

We used flow cytometry to assess the effect of the whole PD-associated microbiome or *K. variicola* on the phagocytosis of Aβ by microglial cells. Microglial cells were seeded into 24-well plates and incubated in RPMI supplemented with 2% FBS and 1% penicillin–streptomycin for 24 h. Bacteria were added at different multiplicities of infection (MOI). After 24 h of co-incubation, 1 µg/mL HiLyte Fluor 488-conjugated Aβ42 (Anaspec, Fremont, USA) was added to the cultures for 2 h. Microglial cells were harvested and washed three times with PBS containing 5% fetal bovine serum (FBS). The final cell suspension was spun down at 200 × g for 5 min; the supernatant was eliminated, and the cells were resuspended in 200 µL of PBS + 5% FBS. Phagocytosis of Aβ42 was measured as the percentage of β-amyloid^+^ microglial cells and analyzed on an Attune™ NxT acoustic focusing cytometer (Invitrogen). Data analysis was done using the FlowJo software.

### TLR-mediated activation of microglial cells

We used two strategies to test the role of TLRs in microglial response to *K. variicola*. After cells were seeded into 24-well plates and incubated in RPMI supplemented with 2% FBS and 1% penicillin–streptomycin for 24 h, first, we targeted TLR2. Microglial cells were pre-treated for 1 h with either TLR2 antagonist (T2.5) at a concentration of 10 µg/mL (InvivoGen) or TLR2 agonist, lipoteichoic acid (LTA-SA) at a concentration of 10 µg/mL (InvivoGen). Then, we targeted TLR9, which is downstream of TLR2. We pre-treated the microglial for 1 h with TLR9 antagonist (ODN2088) at a concentration of 1 µM (InvivoGen) before introducing *K. variicola*. In parallel, we used the TLR9 agonist, CpG oligonucleotide (ODN1826), at a concentration of 1 µM (InvivoGen). These conditions are shown in Additional file 1: Table S3.

### Statistical analysis

All statistical analyses for bone morphometry, relative gene expression, and flow cytometry were performed using *GraphPad Prism Software* version 9.2.0 (GraphPad Software, La Jolla, California, USA). The normality of data distribution was determined using the Shapiro–Wilk test. Outliers were determined using the ROUT method with *Q* = 1% and were excluded. An unpaired *t* test was used to compare two experimental groups. Analysis of variance (ANOVA) followed by multiple comparison Tukey post-hoc tests was applied for three or more groups. Values are expressed as mean ± standard error of the mean. *P* values of < 0.05 were considered statistically significant.

## Results

### Alveolar bone loss and RANKL and OPG expression in ligature-induced periodontal disease

The morphometric analysis confirmed that ligature placement induced progressive PD and bone resorption (Fig. 1B). Bone loss was significantly increased by 25% at day 1 post-ligation compared to baseline (mice without ligatures) (*p* < 0.05). Bone resorption continued to increase over 30 days (*p* < 0.0001). To measure bone turnover, we assessed mRNA expression levels of receptor activators of nuclear factor-kappa B ligand (RANKL) and osteoprotegerin (OPG) in the periodontal tissues. RANKL was significantly upregulated on day 1 and day 10 after ligature placement. While OPG was significantly downregulated on day 1 after ligature placement, its expression showed no difference at the later timepoints compared to the non-ligated baseline. The ratio of RANKL/OPG was increased 1 day after periodontal disease induction (*p* < 0.0001) (Fig. 1C). These data suggested that ligature placement induced progressive periodontal disease and alveolar bone resorption, confirming this preclinical model's acute and chronic inflammatory phases [28].

### Microglial cell response to ligature-induced periodontal disease

**Fig. 2:**
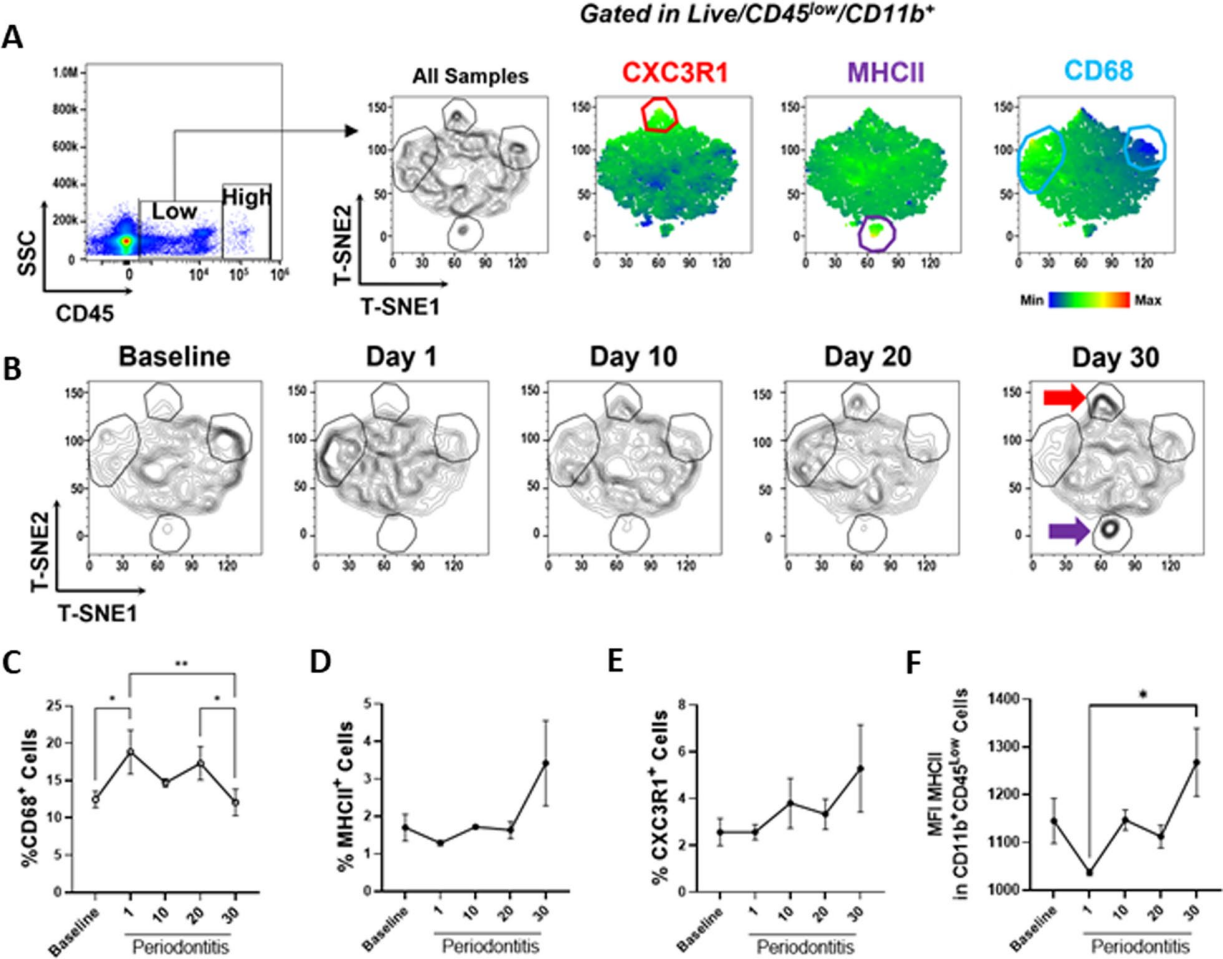
Impact of ligature-induced periodontal disease on microglial cell activation in vivo. **A** Multiparametric analysis of microglia (CD45^low^ CD11b^+^) by flow cytometry, T-SNE plots indicate CX3CR1, MHCII, and CD68 expression on concatenated data (*n* = 3 per group). **B** Differential expression of microglial markers in healthy animals (baseline) and animals with experimental periodontal disease for 1, 10, 20, or 30 days. Red and purple arrows highlight increased expression of CX3CR1 and MHCII, respectively. **C** Percentage of CD68 + (high) **D** MHCII + and **E** CX3CR1 + in microglia. **F** Mean fluorescence intensity (MFI) of MHCII within MHCII + microglial cells. (Mean ± SEM, ANOVA, *p < 0.05, **p < 0.01)

To assess the impact of ligature placement on microglial cell activation in the brain, we performed a multiparametric analysis using flow cytometry for different activation markers of microglial cells (CD45^low^ CD11b^+^) that included fractalkine receptor (CX3CR1), major histocompatibility complex II (MHCII) molecules, and the cluster of differentiation 68 (CD68) (Fig. 2). We observed a time-dependent increase in the percentages of CX3CR1 + and MHCII + cells within the CD45^low^ CD11b^+^ compartment starting at day 10 after ligature placement. The CD68^+^ cells within the CD45^low^ CD11b^+^ population were significantly increased at day 1 post-ligation compared to the baseline group; then, they were significantly reduced at day 30 compared to day 1 and day 20 post-ligation. We also detected a significant increase in mean fluorescent intensity (MFI) of MHCII within MHCII + microglial cells when the experimental periodontal disease was induced for 30 days compared to 1-day post-ligation (*p* < 0.05), collectively suggesting that the periodontal disease led to a significant and early activation of the microglial cells in the brain.

### Inflammatory cytokine production by PD-associated microbiome-stimulated microglial cells

**Fig. 3:**
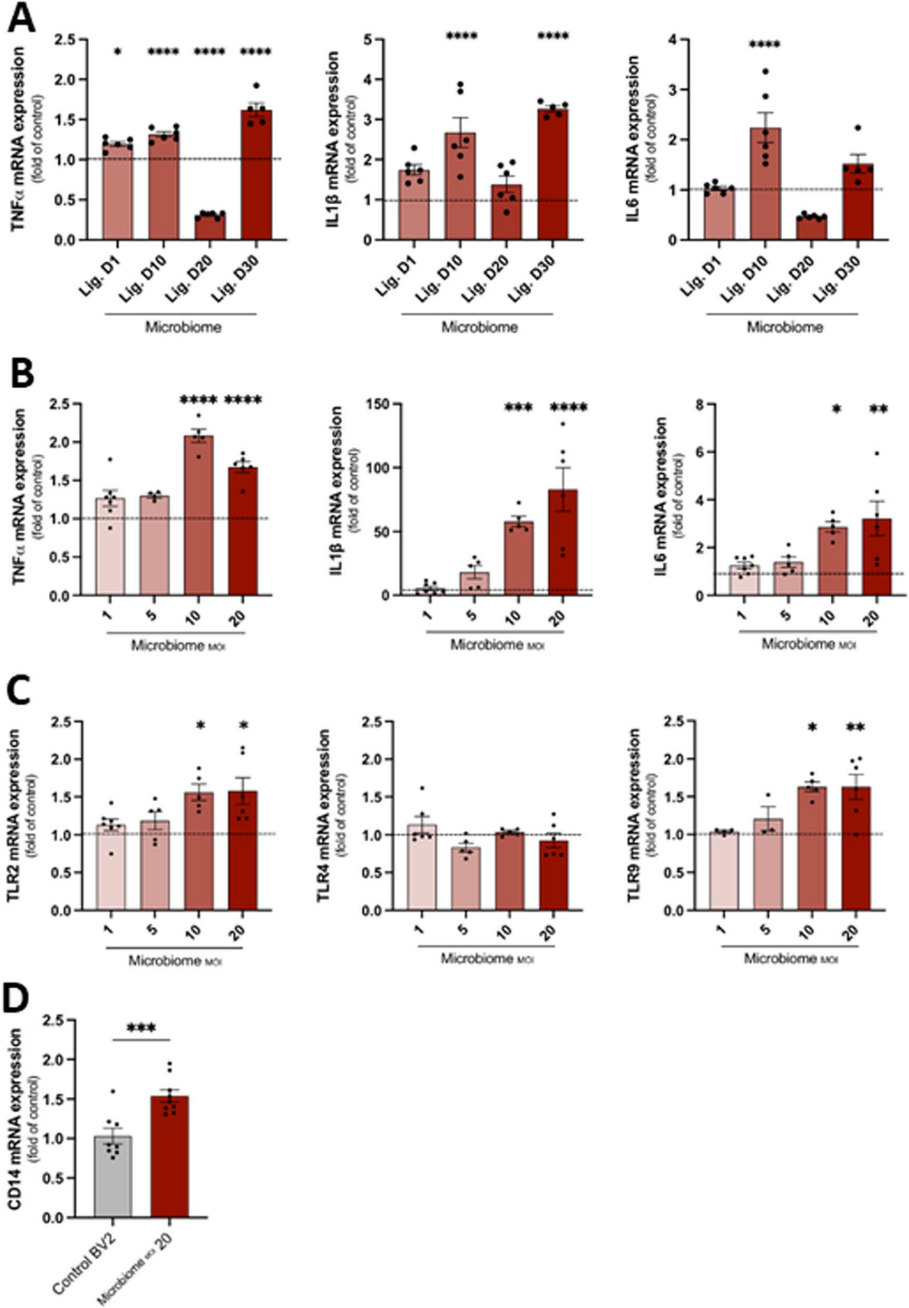
Effect of ligature-induced periodontal disease microbiome on inflammatory cytokines and toll-like receptors (TLRs) expression of microglial cells. **A** Levels of mRNA expression of pro-inflammatory cytokines TNF-α, IL-1β, and IL-6 in microglial cells stimulated with microbiome associated with experimental periodontal disease. Lig. D1, D10, D20, and D30: microglial cells incubated with heat-inactivated PD-associated microbiome harvested from ligatures for different timepoints corresponding to 1,10, 20, and 30 days, respectively, at MOI = 20 for 24 h. (Two different experiments in triplicates, mean ± SEM, ANOVA, *p < 0.05, ****p < 0.0001, all comparisons were made to unstimulated microglial cells represented by the dashed line). **B** Levels of mRNA expression of pro-inflammatory cytokines TNF-α, IL-1β, and IL-6 in microglial cells stimulated with different MOIs of 10-day-old ligature-induced PD-associated microbiome for 24 h. (Three different experiments in duplicates, mean ± SEM, ANOVA, *p < 0.05, **p < 0.01, ***p < 0.001, ****p < 0.0001 all comparisons were made to unstimulated microglial cells represented by the dashed line). **C** Levels of mRNA expression of toll-like receptors TLR2, TLR4, and TLR9 in microglial cells stimulated with different MOIs of 10-day-old ligature-induced PD-associated microbiome for 24 h. (Three different experiments in duplicate, mean ± SEM, ANOVA, *p < 0.05, **p < 0.01, all comparisons were made to unstimulated microglial cells represented by the dashed line). **D** Levels of mRNA expression of co-receptor CD14 in microglial cells stimulated with MOI = 20 of 10-day-old ligature-induced PD-associated microbiome for 24 h (three different experiments in triplicate, mean ± SEM, unpaired *t* test, ***p < 0.001)

We then examined the impact of the heat-inactivated periodontal disease microbiome obtained from the ligatures on the expression of inflammatory cytokines in microglial cells. The PD-associated microbiome collected at different timepoints from ligatures (day 1, day 10, day 20, and day 30) were added to microglial cells. TNF-α, IL-1β, and IL-6, mRNA expression levels, were significantly up-regulated in stimulated microglial cells by 10-day-old PD-associated microbiome compared to unstimulated cells (*p* < 0.0001) (Fig. 3A). Statistically significant increases in TNF-α and IL-1β expression were also observed in 30-day-old PD-associated microbiome compared to unstimulated cells (*p* < 0.0001), suggesting that the PD-associated microbiome directly activated microglial cell function. Then, to determine the optimal level of activation by the microbiome, we treated microglial cells with different MOIs of the 10-day-old PD-associated microbiome (MOI = 1, 5, 10, and 20). MOI of 10 and 20 were at which the PD-associated microbiome activated the microglial function (Fig. 3B). Thus, we used MOI = 20 in our subsequent experiments.

### Toll-like receptor (TLR) and CD14 expression in PD-associated microbiome-stimulated microglial cells

In parallel, we measured mRNA expression levels of TLR2, TLR4, and TLR9. These TLRs are directly involved in bacterial pathogen recognition. TLR4 expression did not show any change in response to PD-associated microbiome. TLR2 and TLR9 expressions were significantly increased in microglial cells stimulated with PD-associated microbiome (Fig. 3C). In parallel, CD14 expression was significantly up-regulated (Fig. 3D), suggesting that TLR2 and TLR9, but not TLR4, were the microglial cell receptors to the mouse PD-associated microbiome, where co-receptor CD14 presented the microbial antigens to the transmembrane TLR2.

### Impact of K. variicola on microglial cells

**Fig. 4:**
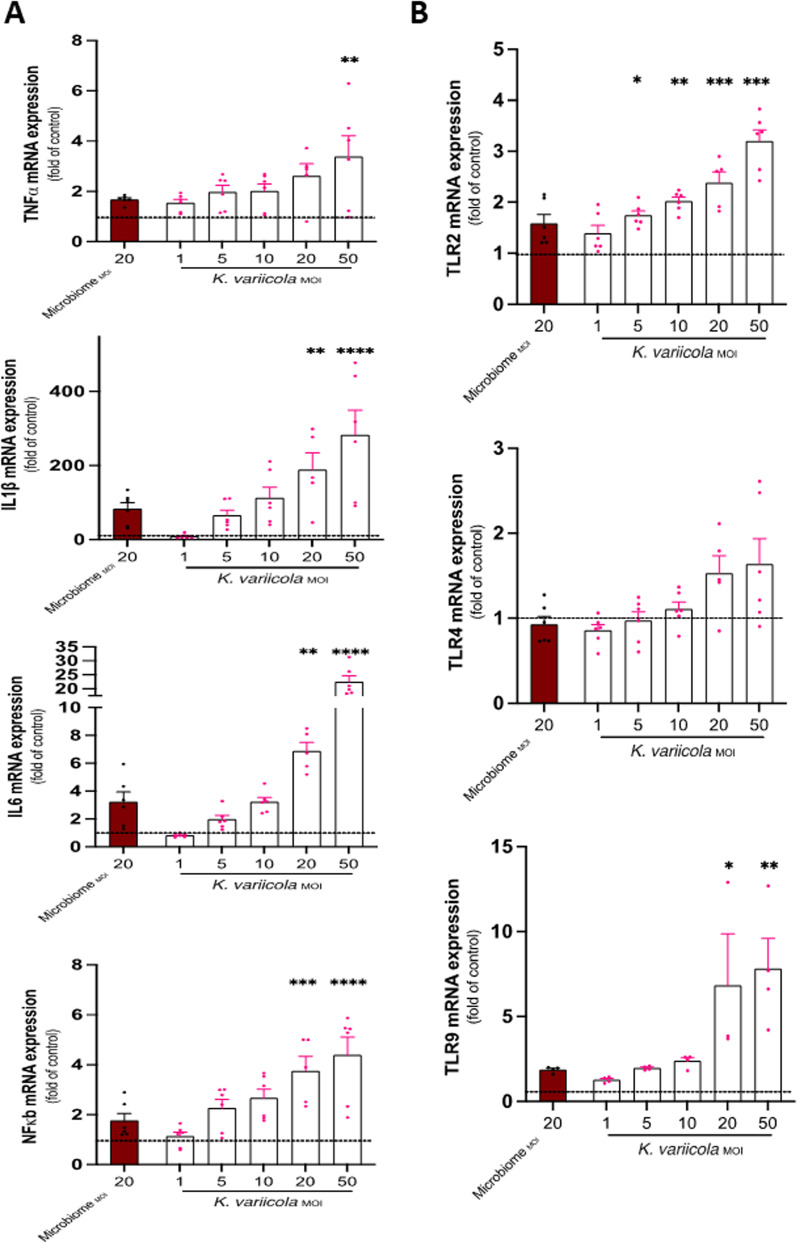
Effect of *K. variicola* on inflammatory cytokines and toll-like receptors (TLRs) expression of microglial cells. **A** Levels of mRNA expression of pro-inflammatory cytokines TNF-α, IL-1β, IL-6, and transcription factor NF-kB in microglial cells stimulated with different MOIs of *K. variicola* for 24 h. **B** Levels of mRNA expression of toll-like receptors TLR2, TLR4, and TLR9 in microglial cells stimulated with different MOIs of *K. variicola* for 24 h. (Three different experiments in duplicate, mean ± SEM, ANOVA, *p < 0.05, ** *p* < 0.01, ***p < 0.001, all comparisons were made to unstimulated microglial cells represented by the dashed line)

We identified *Klebsiella variicola* in the ligatures and brains of 5xFAD transgenic mice, a mouse model of AD, after a 4-week ligature-induced experimental periodontitis through both direct cultures of the brains and 16S rRNA sequencing. Thus, we sought to understand the impact of this specific bacterium detected in the mouse PD model on mouse microglial cell activation. Cells were stimulated with different MOIs of *K. variicola* (1, 5, 10, 20, and 50). IL-1β, IL-6, and NF-kB mRNA expression were significantly and dose-dependently increased after stimulation with *K. variicola* (Fig. 4A). We also measured mRNA expression levels of TLR2, TLR4, and TLR9. While TLR4 expression did not show any difference after stimulation with *K. variicola*, TLR2 and TLR9 mRNA expression were significantly increased in microglial cells (Fig. 4B), suggesting that TLR2 and TLR9, but not TLR4, are the microglial cell receptors that bound to *K. variicola* upon stimulation in line with the impact of whole PD-associated microbiome shown above.

### Impact of TLR2 and TLR9 antagonists on toll-like receptors and inflammatory cytokines mRNA expression in microglial cells

**Fig. 5:**
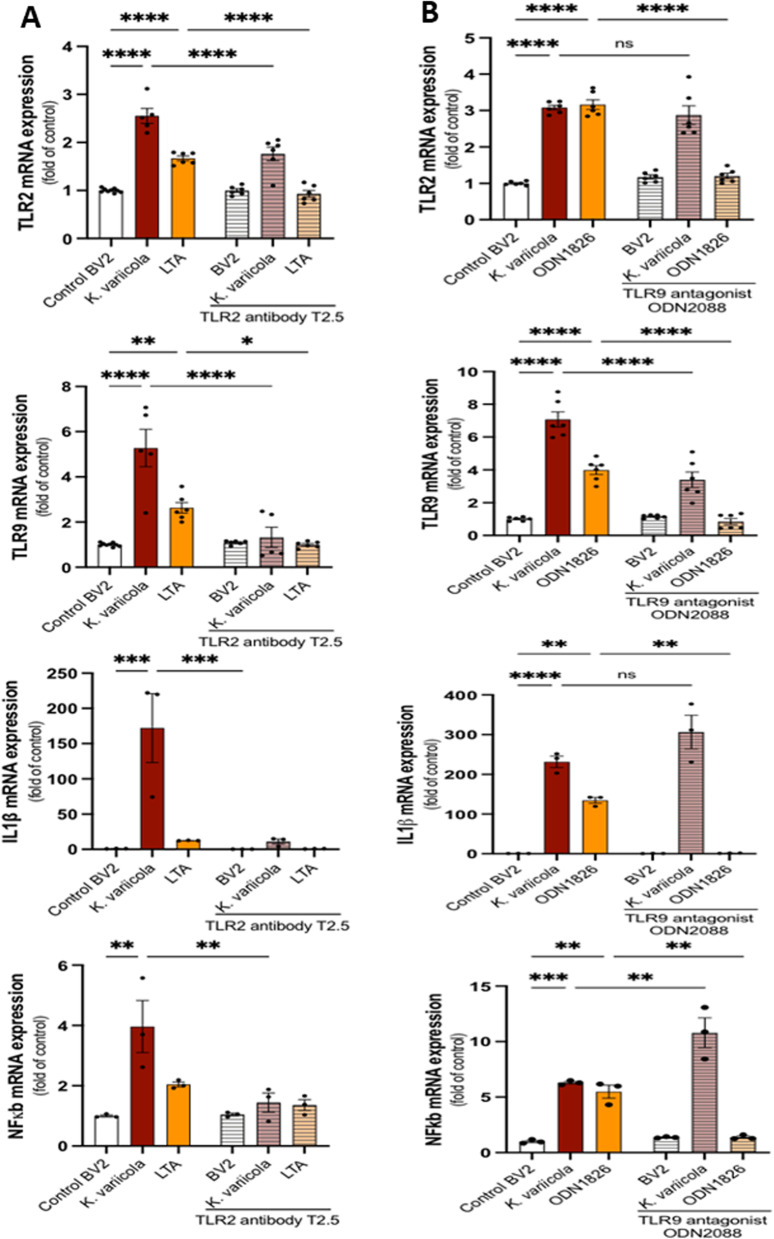
Impact of TLR2 and TLR9 inhibitors on inflammatory cytokines and toll-like receptors (TLRs) expression of microglial cells. **A** Levels of mRNA expression of TLR2, TLR9, IL-1β, and NF-kB in microglial cells stimulated with MOI = 20 of *K. variicola* or Lipoteichoic acid (LTA)–TLR2 agonist at a concentration of 10 µg/mL for 24 h, with or without the addition of TLR2 antagonistic antibody T2.5 at a concentration of 10 µg/mL for 1 h before the *K. variicola* or LTA stimulation. **B** Levels of mRNA expression of TLR2, TLR9, IL-1β, and NF-kB in microglial cells stimulated with MOI = 20 of *K. variicola* or CpG oligonucleotides (ODN1826)–TLR9 ligand at a concentration of 1 µM for 24 h, with or without the addition of TLR9 antagonist (ODN2088) at a concentration of 1 µM for 1 h before the *K. variicola* or ODN1826 stimulation (Mean ± SEM, ANOVA, **p < 0.01, **p < 0.01, ***p < 0.001, ****p < 0.0001).

Since we detected an increased expression for TLR2, TLR9, and pro-inflammatory cytokines in *K. variicola-*activated microglial cells, we used two strategies to test the impact of each TLR agonist and antagonist. First, we targeted TLR2 and used its specific antagonistic antibody T2.5 to block and its specific agonist lipoteichoic acid (LTA) to stimulate TLR2 expressed by microglial cells. LTA-activated microglial cells showed a significant upregulation in TLR2 and TLR9 mRNA expression. This upregulation was lesser than that of *K. variicola-*activated microglial cells (Fig. 5A). When TLR2 antagonist T2.5 was added to microglial cells, it significantly inhibited *K. variicola*-induced expression of TLR2, TLR9, IL-1β, and NF-kB.

Second, we targeted TLR9 and used its specific antagonist (ODN2088) to block and its specific agonist, CpG oligonucleotide (ODN1826), to stimulate TLR9 expressed by microglial cells. ODN1826-activated microglial cells showed a significant upregulation in TLR2, TLR9, IL-1β, and NF-kB expression (Fig. 5B). When TLR9 antagonist ODN2088 was added to microglial cells before their stimulation, it significantly inhibited ODN1826-induced TLR2, TLR9, IL-1β, and NF-kB, while only significantly inhibited *K. variicola*-induced TLR9 (*p* < 0.0001). TLR2 and IL-1β in *K. variicola*-induced microglial cells did not show change with TLR9 antagonist ODN2088 compared to that of *K. variicola*.

### β-Amyloid phagocytosis by microglial cells

**Fig. 6:**
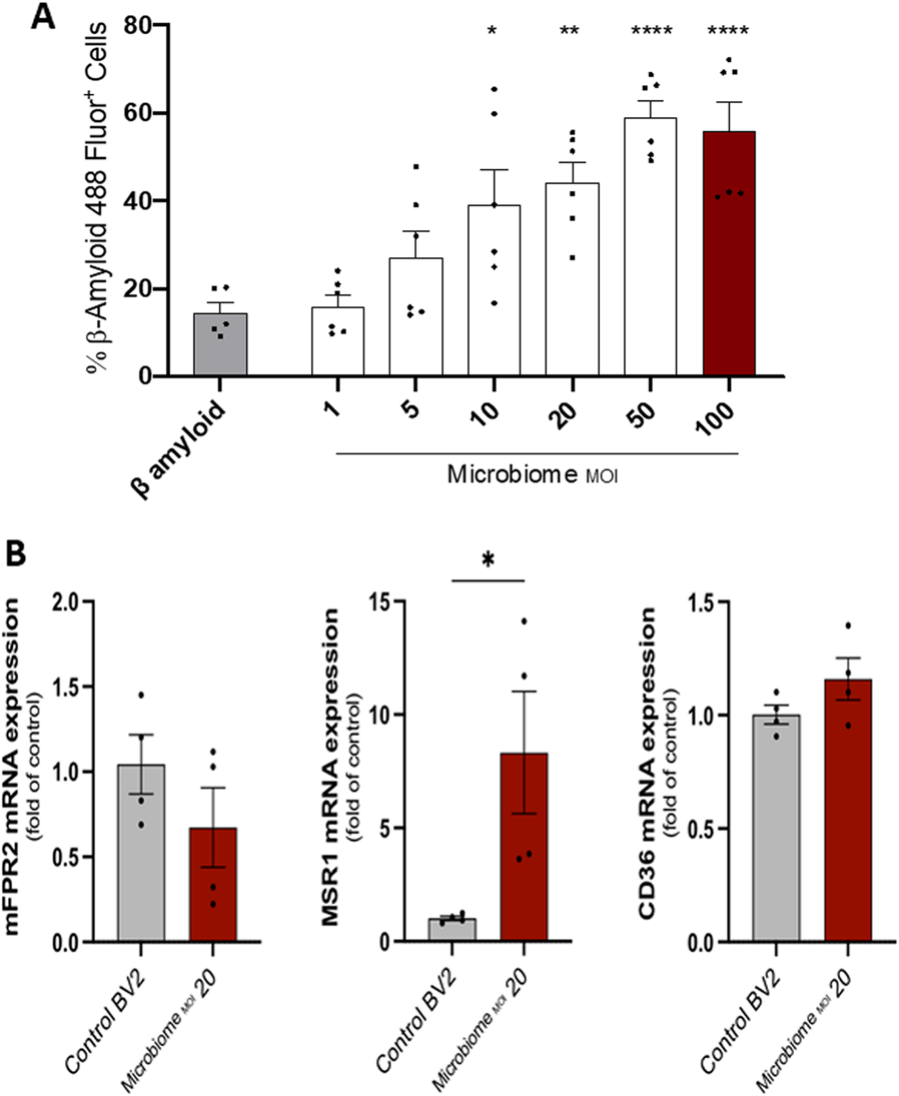
Impact of ligature-induced periodontal disease microbiome on β-amyloid phagocytic capacity of microglial cells. **A** Percentage of β-Amyloid 488 Fluor^+^ microglial cells stimulated with different MOIs of 10-day-old ligature-induced PD-associated microbiome for 24 h. (Experiments in triplicates, mean ± SEM, ANOVA, *p < 0.05, **p < 0.01, ****p < 0.0001 all comparisons were made to unstimulated microglial cells represented by the grey bar). **B** Levels of mRNA expression of mFPR2, class A scavenger receptor MSR1, and Class B scavenger receptor CD36 in microglial cells stimulated with MOI = 20 of 10-day-old ligature-induced PD-associated microbiome for 24 h. (Experiments in duplicate, mean ± SEM, Unpaired *t* test, *p < 0.05)

We then assessed the effect of the PD-associated microbiome on the phagocytic capacity of microglial cells of amyloid β-peptide 42 (Aβ42). Microglial cells were activated with different MOIs of a 10-day-old PD-associated microbiome for 24 h and then incubated with HiLyte Fluor 488-conjugated Aβ42 for 2 h. The percentage of β-Amyloid 488 Fluor^+^ microglial cells was dose-dependently increased with increasing microbiome MOI (Fig. 6A). We then measured the expression of receptors involved in Aβ42 uptake by activated microglial cells. Murine formylpeptide receptor (mFPR2) and Class B scavenger receptor CD36 were not significantly affected by the periodontal disease microbiome. In contrast, class A scavenger receptor (Macrophage scavenger receptor 1 MSR1) expression was significantly increased after stimulation (Fig. 6B).

**Fig. 7:**
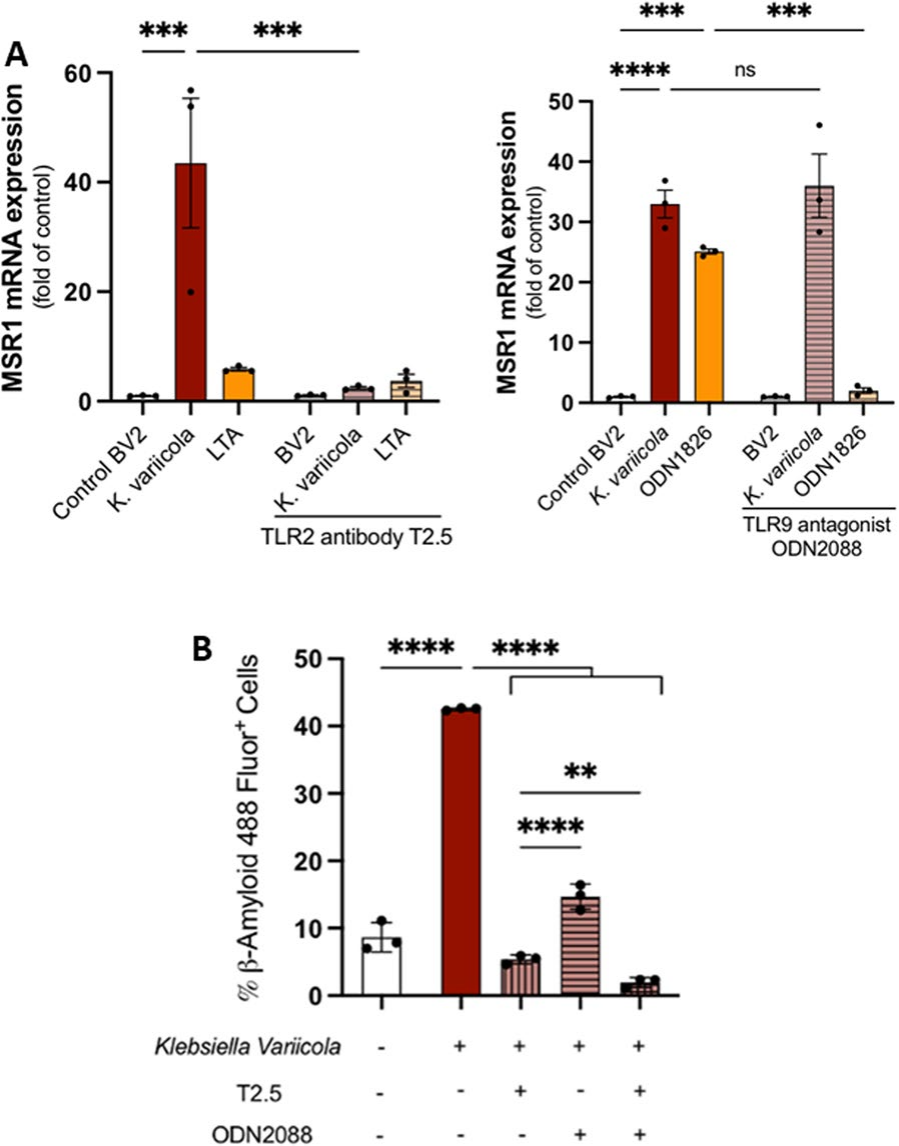
Impact of TLR2 and TLR9 inhibitors on microglial phagocytic capacity of β-amyloid in vitro. **A** Left: level of mRNA expression of MSR1 in microglial cells stimulated with MOI = 20 of *K. variicola* or Lipoteichoic acid (LTA)–TLR2 agonist at a concentration of 10 µg/mL for 24 h, with or without the addition of TLR2 antagonistic antibody T2.5 at a concentration of 10 µg/mL for 1 h before the *K. variicola* or LTA stimulation. Right: level of mRNA expression of MSR1 in microglial cells stimulated with MOI = 20 of *K. variicola* or CpG oligonucleotides (ODN1826)–TLR9 ligand at a concentration of 1 µM for 24 h, with or without the addition of TLR9 antagonist (ODN2088) at a concentration of 1 µM for 1 h before the *K. variicola* or ODN1826 stimulation (Mean ± SEM, ANOVA, ***p < 0.001, ****p < 0.0001). **B** Percentage of β-Amyloid 488 Fluor^+^ microglial cells stimulated with MOI = 20 of *K. variicola* for 24 h with or without the addition of TLR2 antagonistic antibody T2.5 and TLR9 antagonist (ODN2088) either alone or in combination for 1 h before the *K. variicola* stimulation. (a representative experiment in triplicate from three different experiments, mean ± SEM, ANOVA, **p < 0.01, ****p < 0.0001)

*K. variicola* also significantly upregulated MSR1 expression (Fig. 7A). TLR2 antagonistic antibody T2.5 significantly inhibited *K. variicola*-induced MSR1. MSR1 did not show any difference with TLR9 antagonist ODN2088.

*K. variicola* increased the phagocytosis of Aβ42 by the microglial cells (Fig. 7B). TLR2 antagonistic antibody T2.5 and TLR9 antagonist (ODN2088) were added either alone or in combination before the *K. variicola* stimulation. TLR2 and TLR9 inhibition significantly reduced the percentage of β-Amyloid 488 Fluor^+^ cells. There was a significant reduction of Aβ42 uptake in *K. variicola*-stimulated microglial cells with TLR2 antagonistic antibody T2.5 compared to TLR9 inhibition (*p* < 0.0001). Combined inhibition of both TL2 and TLR9 showed a further decrease of Aβ42 phagocytosis in *K. variicola*-stimulated cells compared to each inhibition alone (Fig. 7B).

## Discussion

Microglial cells are critical for the neuroinflammatory process within the brain [1, 2, 29]. In neurodegenerative diseases, where neuroinflammation is a part of the pathogenesis, such as AD, microglial cells become chronically activated, releasing pro-inflammatory cytokines and displaying abnormal phagocytic capacity of proteins, such as Aβ peptides, resulting in further microglial activation [1, 2, 30]. Microbial components can trigger microglial activation and initiate the neuroinflammatory process. Recent studies have suggested that oral microbes and/or their virulence factors may participate in this process [22, 23, 31], thus mechanistically linking oral health status with neuroinflammation risk. Periodontitis-associated microorganisms have been found in the brains of AD patients; however, a direct interaction between putative periodontal pathogens, their virulence factors, and microglia remains unknown. In the present study, we described the mechanistic link between periodontal disease and associated pathogens and microglial activation. We used a preclinical model of ligature-induced periodontal disease, which initiates the inflammatory response by generating microbial dysbiosis due to plaque accumulation around the maxillary molars, mimicking the etiopathogenesis of the disease in humans. We observed alveolar bone loss and an enhanced microglial activation phenotype in the brains of wild-type mice that become more prominent as the days with ligatures increase. We corroborated these in vivo findings using an in vitro approach to evaluate the direct impact of the ligature-induced PD-associated microbiome on the activation and phagocytic capacity of microglial cells, a mouse microglial cell line. Microglial cells were stimulated with a mouse PD-associated microbiome; we found that they were activated and able to mount a pro-inflammatory immune response through TLR2 and TLR9 activation upon microbial stimulation. We also detected enhanced phagocytosis of Aβ in activated microglia through MSR1 overexpression, which was reduced using TLR2 and TLR9 inhibitors. The data from this study demonstrated a direct and specific receptor-mediated impact of the PD-associated microbiome on microglial cell activation.

Neuroinflammation is a vital and early component of neurodegenerative diseases. In AD pathogenesis, for instance, increased microglial activation is detected before detecting Aβ plaques and neurofibrillary tau tangles, the specific hallmarks of the disease [30]. Our data showed that microglial cells within the adult brain, defined as CD11b^+^ and CD45^low^ [32], showed an increase in the expression of different activation markers, such as fractalkine receptor (CX3CR1) and major histocompatibility complex II (MHCII), that started on day 10 after ligature placement. Our data also demonstrated that the mean fluorescent intensity of the activation marker MHCII within the MHCII + microglial cells compartment was significantly higher at day 30 than on day 1 post-ligature placement. A previous study showed an increase in MHCII + microglial cells accompanied by neuron loss, neuroinflammation, and infiltration of other immune cells in a rat model of Parkinson's disease [33]. The fluctuation of CD68^+^ cell percentage within the CD45^low^ CD11b^+^ population after periodontal disease induction could be attributed to the fact that the CD68 receptor level is strongly up-regulated during inflammation and can internalize from the cell surface to endosomes immediately after stimulation [3]. Subsequently, these chronically activated microglia may strongly drive neuroinflammation initiation.

To explore the possible pathways involved in microglial activation during the progression of PD, we used an innovative approach in our in vitro experiments, where we co-cultured a mouse microglial cell line with the mouse PD-associated microbiome and its virulence factors harvested from the ligatures. Previous studies used microorganisms in the human periodontitis microbiome and specific bacterial cell components to infect mouse microglial cells and achieve microglial activation or induce experimental periodontitis in mice [34–37]. For example, a previous in vitro study demonstrated that rat microglial cells stimulated with *Aggregatibacter actinomycetemcomitans* LPS highly expressed the same inflammatory cytokines and toll-like receptors TLR2 and TLR4 that we found in the present study [34]. Those studies, while providing useful data for understanding the potential impact of human bacteria in the neuroinflammatory processes, could be more extensive in their translational value as they represent interspecies findings between mice and humans. To overcome this limitation, we used a murine model of experimental periodontal disease, identified the periodontal microbiome associated with disease in mice, and used these specific bacterial species to activate the murine microglial cells. Until this study, this model has not yet been reported. The data showed that the PD-associated microbiome directly activated microglial cells and increased the expression of pro-inflammatory cytokines TNF-α, IL-1β, and IL-6. Furthermore, we identified that TLR2, TLR9, and co-receptor CD14 were highly expressed during this activation showing the specific TLR-mediated pathway of microglial activation in response to PD-associated microorganisms in the same mammalian species.

Our data suggested that the interaction of microglia with the PD-associated microbiome had a significant role in shaping microglial functions. While the binding of PD-associated microorganisms to TLR2 and TLR9 on microglia increased their inflammatory cytokine expression, it also increased the phagocytosis of Aβ42. This finding was in line with previous studies, where activation of TLR2 on microglia by its corresponding ligand peptidoglycan from *Staphylococcus aureus* led to NF-κB activation and enhanced microglial chemotactic response and phagocytic capacity of Aβ42 [35]. Another in vitro study demonstrated that the activation of TLR9 expressed on microglia by CpG-containing ODN increased their ability to endocytose Aβ42 [37]. It is critical to note that our in vitro microglial activation with PD-associated microbiomes was done for 24 h, considered an acute model of microglial activation as previously suggested [38], and may not fully represent the chronic or evolving microglial activation that happens in AD [39]. Nevertheless, the TLR2/TLR9-mediated pathway underlines the microglial response to microorganisms in this context, PD-associated microbiome.

In a parallel experiment, we identified bacteria of the genus *Klebsiella* to be prominent in the microbiome of mice after experimental PD-associated microbial colonization of both ligatures and brain specimens. The direct brain cultures and 16S rRNA sequencing showed that the species was *K. variicola,* in line with a recent study, which also detected this bacterial species in the oral cavity of mice after experimental periodontitis induction [40]. Therefore, we used this specific species to explore its impact on microglial cell activation and function. Our data confirmed that *K. variicola* was able to activate microglial cells directly and up-regulate the expression of pro-inflammatory cytokines TNF-α, IL-1β, and IL-6, similar to the ligature microbiome-induced microglial activation but with higher immunogenic reactivity, suggesting that this PD-associated microorganism may have unique virulence factors and could promote more neuroinflammation. Furthermore, our data identified that TLR2 and TLR9 were highly expressed in microglia during this activation. We also detected increased Aβ42 phagocytosis by the activated microglia mediated by MSR1 overexpression. Since we identified that TLR2 and TLR9 expressions were upregulated upon microglial stimulation with *K. variicola*, we aimed to explore the impact of their inhibition using TLR2 antagonistic antibody T2.5 and TLR9 antagonist (ODN2088) before microglial activation with *K. variicola*. Our data showed that inhibition of TLR2 caused downregulation in the expression of TLR2, TLR9, and IL-1β. However, the inhibition of TLR9 caused downregulation in the face of TLR9. Thus, *K. variicola* was still able to mount pro-inflammatory microglial activation through TLR2. A possible explanation for this finding is that TLR2 is found on the cell surface and is required for optimal microorganism-induced phagocytosis by innate immune cells; consequently, phagocytosis is a necessary pre-requisite for TLR9 identification of bacterial genomic DNA in late endosomal compartments. Our data also suggested that the inhibition of TLR2 and TLR9 abrogated Aβ42 internalization by activated microglia. These data collectively suggested the imperative role of TLR2 and TLR9 in microglial activation and their subsequent Aβ42 phagocytic function mediated by the PD-associated microbiome.

It is important to note that we did not use any non-*K. variicola* control, because our line of thought was to (1) induce periodontal disease by placing ligatures around maxillary molars at different timepoints, (2) retrieve the ligatures, (3) collect the total PD-associated microbiome from the ligatures, (4) identify the live bacteria by culture and sequencing, (5) use the entire microbiome to stimulate microglial cells, and (6) use *K. variicola* that was the dominant bacterial species in the microbiome to stimulate microglial cells. Therefore, we did not randomly choose *K. variicola* as a pathogen; we identified *K. variicola* as a part of the total murine periodontal microbiome and compared its effects to the entire microbiome.

One limitation of the current study is that we could not have microbiomes associated with healthy periodontal tissues to use as controls, since the collection method was the ligature placed around the teeth, where the oral microbiome colonized as a part of the PD process. Since placing the ligatures required at least 1 day to colonize bacteria and subsequently induce PD and inflammation, it was impossible to obtain a true baseline. Changing the sampling methodology, such as using oral swabs, will reflect a different microbial population than these would be from everywhere in the mouth of a mouse [41]. Thus, the ligature-associated PD microbiome reflected the actual periodontal microbiome gathering around the teeth and was responsible for periodontal disease and inflammation.

**Fig. 8:**
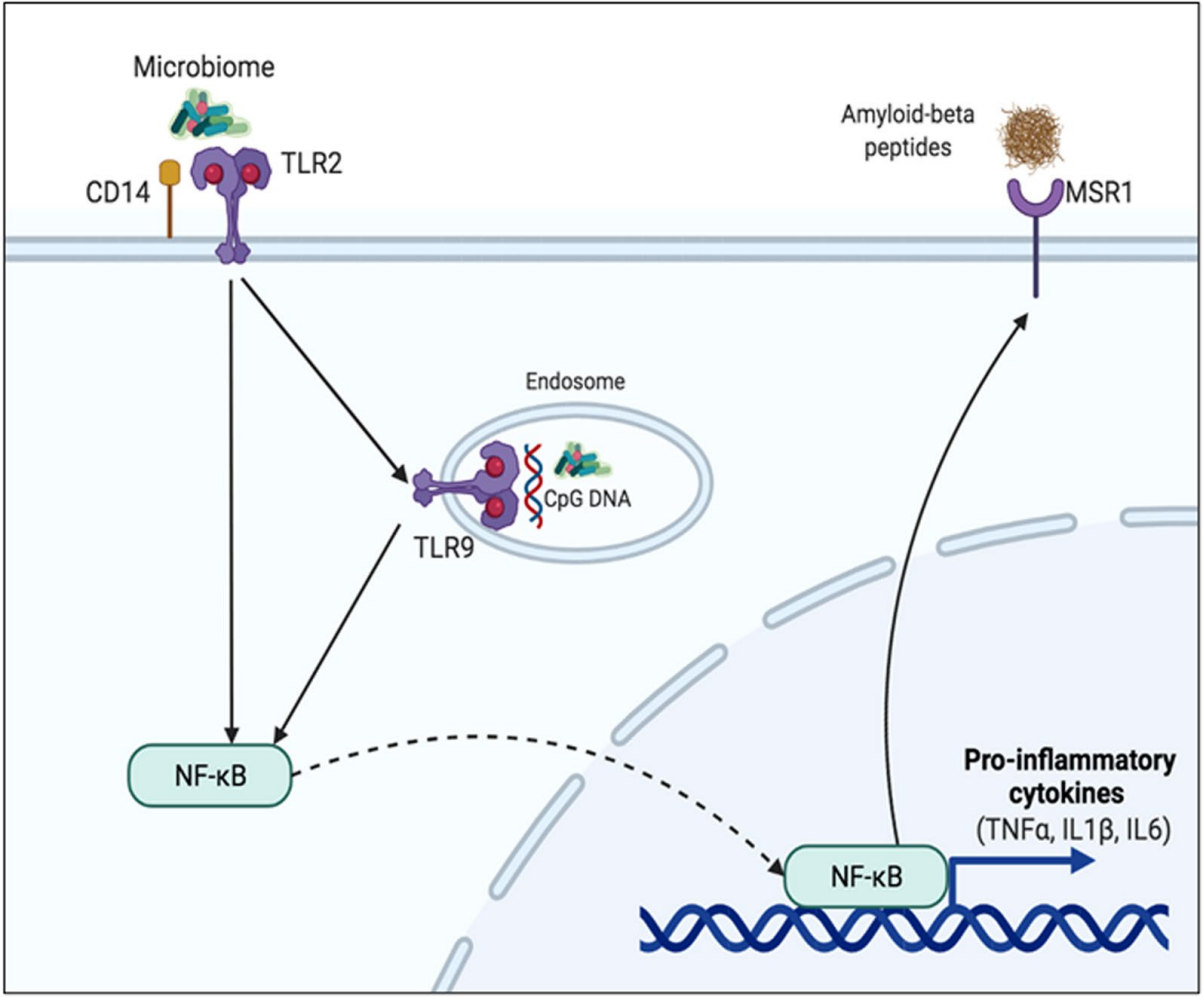
Schematic diagram of TLR2 and TLR9 activation and microglial expression of pro-inflammatory cytokines and macrophage scavenger receptor1 (MSR1). TLR2 and TLR9 on microglia upon activation by microbiome associated with periodontal disease activate NF-κB. The subsequent activation of NF-κB increases the transcription of the pro-inflammatory cytokines and MSR1. In addition, the MSR1 expressed by activated microglia mediates cell phagocytosis of Aβ42

This study established a clear relationship between the PD induced by ligature placement and microglial cell functions and markers of neuroinflammation. Our findings may be relevant to neurodegenerative disorders, where neuroinflammation plays a role. In conclusion, the data demonstrated that periodontal disease activated microglial cell activation at multiple levels, as shown in the model (Fig. 8). The microbiome associated with PD directly stimulated the microglial cell through TLR2 and TLR9, further activating NF-κB. The subsequent activation of NF-κB increased the transcription of the pro-inflammatory cytokines and MSR1. In addition, the MSR1 expressed by activated microglia-mediated cell phagocytosis of Aβ42 demonstrates the impact of periodontal infection on the brain microglial cells, which may contribute to the initiation and progression of the neuroinflammation in neurodegenerative processes. Clinical implications and translation of these findings to humans are high. In humans, LPS and gingipains of *P. gingivalis* (a major periodontopathogen linked to several systemic diseases) were already demonstrated in the brains of AD patients [20, 21]. Our work demonstrates a clear mechanistic role of oral pathogenic bacteria activating the microglial cells in the same mammalian species and mimicking the etiopathogenesis of the disease in humans. Because bacteria colonizing the brain as a result of PD would further increase the recruitment of peripheral phagocytes and chemokines across an impaired and “leaky” blood–brain barrier due to AD, identifying the impact of oral bacteria on the brain cells and neuroinflammation is critical for developing targeted therapies and preventing infecto-inflammatory processes that exacerbate AD-associated pathologies in humans, which need to be tested in clinical trials.

## Supplementary Information

**Additional file 1: Table S1.** List of TaqMan Gene Expression Assays used for cDNA amplification by qPCR. ** Table S2.** List of antibodies used for flow cytometry. **Table S3.** List of TLR agonists and antagonists used.

## Data Availability

The original contributions presented in the study are included in the article and supplementary material. Further inquiries can be directed to the corresponding author.
