# Supplementary material for: Microglial cell response to experimental periodontal disease
Source: J Neuroinflammation. 2023 Jun 14;20:142. doi: 10.1186/s12974-023-02821-x (PMC10265806; doi:10.1186/s12974-023-02821-x)
Supplement: Supplementary file 1 — Additional file 1: Table S1. List of TaqMan Gene Expression Assays used for cDNA amplification by qPCR. Table S2. List of antibodies used for flow cytometry. Table S3. List of TLR agonists and antagonists used. [file 12974_2023_2821_MOESM1_ESM.docx]

**Additional tables**

Table S1. List of TaqMan Gene Expression Assays used for cDNA amplification by qPCR

| **Target gene** | **Assay ID** |
| --- | --- |
| β-actin | Mm00607939_s1 |
| CD14 | Mm01158466_g1 |
| CD36 | Mm00432403_m1 |
| CtsK | Mm00484039_m1 |
| IL-1β | Mm00434228_m1 |
| IL-6 | Mm00446190_m1 |
| IL-10 | Mm01288386_m1 |
| mFPR2 | Mm00484464_s1 |
| MSR1 | Mm00446214_m1 |
| NF-kB | Mm00476361_m1 |
| OPG (Tnfrsf11b) | Mm00435452_m1 |
| RANKL (Tnfsf11) | Mm00441906_m1 |
| TNF-α | Mm00443258_m1 |
| TLR2 | Mm01213946_g1 |
| TLR4 | Mm00445273_m1 |
| TLR9 | Mm07299609_m1 |

Table S2: List of antibodies used for flow cytometry

| **Antibody** | **Conjugate** | **Clone** | **Dilution** | **Catalog No.** | **Source** |
| --- | --- | --- | --- | --- | --- |
| Anti-CD45 | PE | 30-F11 | 1:200 | 103105 | Biolegend |
| Anti-CD11b | PerCP/Cyanine5.5 | M1/70 | 1:200 | 101227 | Biolegend |
| Anti-CD68 | Brilliant Violet 421 | FA-11 | 1:100 | 137017 | Biolegend |
| Anti-MHCII | APC | M5/114.15.2 | 1:200 | 107613 | Biolegend |
| Anti-CX3CR1 | Brilliant Violet 711 | SA011F11 | 1:100 | 149031 | Biolegend |

Table S3: *List of TLR agonists and antagonists used*

|  | **Agonist** | **Catalog code** | **Antagonist** | **Catalog code** |
| --- | --- | --- | --- | --- |
| **TLR2** | LTA-SA | tlrl-slta | MAb mTLR2 (T2.5) | mab2-mtlr2 |
| **TLR9** | ODN1826 | tlrl-1826 | ODN2088 | tlrl-2088 |
